# Ozone rectal insufflation inhibits the development of atherosclerosis in ApoE−/−mice, which is mediated by the regulation of gut microbiota and metabolites

**DOI:** 10.3389/fmicb.2025.1597695

**Published:** 2025-06-25

**Authors:** Ruihua Li, Yulin Wang, Xuelian Ji, Qi Han, Kang He, Haobin Zhao, Hongbo Li

**Affiliations:** ^1^Weifang People's Hospital, Shandong Second Medical University, Weifang, China; ^2^Department of Anesthesiology, The First Affiliated Hospital of China Medical University, Shenyang, China

**Keywords:** atherosclerosis, ozone rectal insufflation, gut microbiota, gut microbiota-derived metabolites, short-chain fatty acids

## Abstract

**Introduction:**

Atherosclerotic cardiovascular disease is the world’s leading cause of death. Researches have proven that ozone therapy can effectively inhibit the development of atherosclerosis; however, the underlying mechanisms remain unclear. This study aims to explore how ozone rectal insufflation (O3-RI) prevents atherosclerosis.

**Methods:**

O3-RI therapy involves administering medical ozone via rectal injection to prevent multiple diseases. ApoE−/− mice were fed a high-fat diet (HFD) to induce atherosclerosis. Gut microbiota was identified by 16S rRNA sequencing. Microbial metabolites were determined by liquid chromatography-mass spectrometry(LC–MS).

**Results:**

O3-RI reduced serum LDL-C levels by approximately 29.6% and decreased both atherosclerotic lipid areas and plaque area in ApoE−/− mice. Additionally, O3-RI improved gut microbiota imbalance caused by a high-fat diet in these mice. Notably, O3-RI increased beneficial microorganisms like *Lactobacillus* and *Bifidobacterium*, which help reduce atherosclerosis. Additionally, O3-RI also increased the levels of short-chain fatty acids, such as propionic acid and butyric acid, At the same time, it decreased harmful microbial metabolites, like TMA and TMAO in fecal samples.

**Discussion:**

This research indicates that O3-RI effectively inhibits atherosclerosis and reduces serum LDL-C levels. These effects are likely linked to ozone’s optimization of gut microbiota and regulation of microbial metabolites in ApoE−/− mice. The non-invasive O3-RI may play a crucial role in managing and treating atherosclerosis. The non-invasive O3-RI may play a crucial role in the managing and treating of atherosclerosis.

## 1 Introduction

Cardiovascular disease (CVD) is the leading cause of death worldwide, accounting for approximately one-third of the yearly deaths of 17.9 million people (Fan and Watanabe, 2022; Zhou et al., 2019). Atherosclerosis is the leading cause and the primary pathological process of CVD (Zhang et al., 2021). Atherosclerosis is a chronic disease influenced by multiple factors including dyslipidemia, abesity, hypertension, diabetes, smoking, alcohol consumption, and high homocysteine levels (Herrington et al., 2016; McCully, 2015). Atherosclerosis treatment primarily includes pharmacological and non-pharmacological methods. Pharmacological therapy is the mainstay of treatment for atherosclerosis. However, their side effects limit their use to a certain extent. Some non-pharmacological therapies are often costly, invasive, and their effectiveness is often nonspecific, which leads to lower acceptance. Thus, developing new treatment methods to slow atherosclerosis progression and lower cardiovascular disease rates has become a priority. Recent studies have shown that ozone (O3) therapy can effectively prevent the development of atherosclerosis (Delgado-Roche et al., 2013; Zhang et al., 2014). Ozone is a volatile gas composed of three oxygen atoms and has limited solubility in water. Ozone was widely used to injuries sustained by soldiers during the First and Second World Wars (Thompson, 1859; Xie et al., 2016). Ozone therapy involves a combination of oxygen and ozone delivered through methods such as autologous blood transfusion, ozonate water, ozone gas baths, and ozone rectal insufflation (O3-RI). Due to its antioxidant, anti-inflammatory, and antiapoptotic properties, ozone therapy is used to treat a range of conditions, including ischemic diseases, infections, age-related macular degeneration, neurological degenerative disorders, sleep disorders, COVID-19 and metabolic diseases (Bocci et al., 2009; Masan et al., 2021; Setyo Budi et al., 2022; Yan et al., 2022). While some studies suggest that ozone may be effective in treating atherosclerosis, the exact mechanism is still unclear.

Thousands of species and billions of microorganisms are symbiotic in the gut (Duttaroy, 2021). The gut microbiome can regulate the physiological functions of various systems in the whole body (Alexandrescu et al., 2024). The role of the interaction between gut and other organs in diseases is constantly being explored (Dumitru et al., 2024). Recent research has shown that the composition of gut microbiota are closely related to the development of atherosclerosis (Verhaar et al., 2020; Witkowski et al., 2020). Some studies have pointed out that gut microbiota is related to the formation of atherosclerotic plaques in ApoE−/− mice (Stepankova et al., 2010). Metagenomic analyses have shown that the stability of atherosclerotic plaques is associated with microbial composition in atherosclerosis (Sanchez-Rodriguez et al., 2020). The gut microbiome is related to the host’s inflammatory status, and the gut microbiome of patients with symptomatic atherosclerosis exhibits characteristic changes. Analysis of the gut microbiome in patients with atherosclerosis reveal an increase in microorganisms that produce pro-inflammatory peptidoglycans, while those produce anti-inflammatory carotenes are decreased (Karlsson et al., 2012). The increase in the abundance of anti-inflammatory genera, such as *Bifidobacterium* and *Roseburia* can slow down the progression of atherosclerosis (Alexandrescu et al., 2024).

Numerous studies have shown that the anti-atherosclerotic effects of various substances are mediated by the regulation of gut microbiota (Yang et al., 2016; Zhu et al., 2018). Trimethylamine is an important diet-induced metabolite produced by the gut microbiota. Trimethylamine(TMA) can be catalyzed to produce Trimethylamino-N-oxide (TMAO) via hepatic metabolism. High levels of TMAO in plasma can predict the risk of long-term adverse events in patients with atherosclerotic peripheral arterial disease. Many fundamental studies have confirmed that TMAO can alter cholesterol metabolism, promote inflammation, cause endothelial dysfunction, and activate platelets. It also directly contributes to the occurrence and progression of atherosclerosis (Canyelles et al., 2023; Papandreou et al., 2020). Short-chain fatty acids (SCFAs) are mainly derived from the fermentation of dietary fibre by gut microbiota. Some studies indicate that SCFAs significantly impact atherosclerosis by regulating Treg cell production, inhibiting histone deacetylases (HDACs), and affecting lipid metabolism. Some studies indicate that SCFAs significantly impact atherosclerosis by regulating Treg cells production, inhibiting histone deacetylases (HDACs) and affecting lipid metabolism (Arpaia et al., 2013; Tayyeb et al., 2019). Butyrate inhibits endothelial NOX2 and ROS expression through the PPARδ/miR-181b pathway, thereby improving endothelial function and preventing the development of atherosclerosis. Butyrate inhibits endothelial NOX2 and ROS expression through the PPARδ/miR-181b pathway, thereby improving endothelial function and preventing the development of atherosclerosis (Tian et al., 2021). These results suggested that SCFAs play a variety of roles in different pathophysiological stages of atherosclerosis. Understanding the relationship between gut microbiota-derived metabolites and atherosclerosis can improve our insight into cardiovascular disease susceptibility and identify potential therapeutic targets.

O3-RI is a treatment method that involves of injecting ozone into the rectum to treat and prevent diseases. The effect of O3-RI stem from ozone’s influence on the intestinal tract, it has been suggested that ozone can act both locally and systematically, impacting diseases by regulating the structure and composition of gut microbiota (Hidalgo-Tallón et al., 2013; Himuro, 2018). Can O3-RI inhibit the progression of atherosclerosis by altering gut microbiota and metabolites? Currently, there are no reports regarding ozone’s effects on the progression of atherosclerosis through regulating gut microbiota. This study aimed to clarify the inhibitory role of O3-RI on atherosclerosis, specifically focusing on its association with the regulation of gut microbiota and metabolites in ApoE−/− mice.

## 2 Materials and methods

### 2.1 Atherosclerotic animal

This study was approved by the Ethics Committee of Shandong Second Medical University and performed in accordance with the US National Institutes of Health Guide for the Care and Use of Laboratory Animals. The male ApoE−/− mice on a C57BL/6 background (6–8 weeks old, 18–22 g body weight) were purchased from Charles River Laboratory Animal Co., Ltd. (Beijing, China). The animals were kept in standard mouse cages, with six mice per cage. They had free access to food and water and were under a 12-h light/dark cycle at a temperature of 23 ± 1°C and humidity levels of 55–60%.

After a 2-week acclimatization period, the ApoE−/− mice were randomly divided into three groups (*n* = 6 in each group): a normal diet (ND)group, a high-fat diet (HFD)group, and HFD group with ozone therapy (HFD + O3). The HFD included basal feed, 21% lard, and 0.15% cholesterol (Beijing Keao Xieli Feed Co., Ltd., China). All mice were fed their designated diets for a total of 12 weeks. In the fifth week, the HFD + O3 group and HFD group, respectively, received ozone therapy and air therapy every other day, both administered through rectal insufflation (Ozone Therapy Devices, Kastner-Praxisbedarf GmbH). After 8 weeks of treatment, we recorded body weight, food consumption, behavior, and coat color every 2 weeks. The mice were anaesthetized with 1% sodium pentobarbital and sacrificed to measure aortic lesion size. In addition, tissues and blood samples were collected and kept at −80°C for further analysis.

### 2.2 O3-RI treatment

In our study, gaseous ozone was generated using an ozone therapy device (Kastner-Praxisbedarf-GmbH, Rastatt, Germany), with a concentration of 20 μg/ml and a does of 1 mg/kg(bw), based on prior studies by Cheng et al. (2024), that demonstrated efficacy and safety under similar conditions. After immobilizing each mouse, a 0.1 mm × 5 cm antioxidant cannula was carefully inserted into the colorectum, reaching the colon transversum. Ozone was introduced through the cannula into the mice. To prevent gas leakage, the mice’s anus was gently sealed for 1 to 2 min after treatment. The HFD group was immobilized and received air as placebo, while the ND group was immobilized without any additional treatment.

### 2.3 Biochemical analysis

Blood samples were harvested from the left ventricle of mice, and serum was obtained by centrifugation. Total cholesterol (TC), triglycerides (TG), low-density lipoprotein cholesterol (LDL-C), and high-density lipoprotein cholesterol (HDL-C) were measured using commercial kits (Rayto Life and Analytical Sciences Co., Ltd. Shenzhen, China. S03042, S03027, S03029 and S03025, respectively) following the manufacturer’s instructions.

### 2.4 Oil-red O staining

The mice were anaesthetized and then perfused using 4% paraformaldehyde. The aortic tissues, collected from the aortic sinus to the abdominal aorta, were fixed in 10% formaldehyde. They were dehydrated in a sucrose solution, embedded in optimum cutting temperature compound (OCT), cut into 8–10 μm sections, and stored at −20°C for Oil red O staining. Tissue sections were stained with Oil red O (Servicebio, Wuhan, China. G1015) for 8 to10 minutes, followed by staining with hematoxylin (G1004 Servicebio) for 3to 5 min. Atherosclerotic lipid areas appeared as red-stained areas and were quantified using Image J software (V1.8.0).

### 2.5 Hematoxylin and eosin (HE) staining

Aortic tissues fixed with paraformaldehyde were embedded in paraffin and sliced into 4 μm sections. The paraffin sections were placed on glass slides for dewaxing and hydration. They were then stained with hematoxylin (G1003 Servicebio, Wuhan, China) for 3 to 5 min. Immediately after, the paraffin sections were transferred to the differentiation solution for a few seconds to 30 s and then washed with water. Next, sections were stained with eosin (G1003 Servicebio, Wuhan, China) for 5 min. After washing, the stained aortic tissue sections were dehydrated and sealed. Examined the stained sections under a microscope and captured images to analyze the staining pattern. The area of atherosclerotic plaque was defined as the difference between the total vessel area and the lumen area.

### 2.6 16S rRNA-sequencing

Total DNA was extracted from the faecal samples (ND, HFD, and HFD + O3 groups) using the E.Z.N.A.® soil DNA Kit (Omega Bio-Tek, Norcross, GA, U.S.) according to the manufacturer’s instruction. The hypervariable V3-V4 region of the bacterial 16S rRNA gene was amplified using the T100 Thermal Cycler PCR thermocycler (BIO-RAD, U.S.), The V3-V4 region has a length of approximately 460 bp, the V3 region adds approximately 100 bp compared to V4 region, potentially enhancing resolution for certain bacterial genera. The PCR products were exacted, purified and quantified. The purified PCR products were constructed and sequenced using the Illumina PE300/PE250 platform (Illumina, San Die-go, U.S.). To ensure data comparability at the same sequencing depth, we performed random rarefaction to 95% of the lowest sequence amount. Fastp software was used to filter the quality of original double-ended sequencing data, and FLASH software was employed to merge the filtered sequences. The optimized sequences were then clustered and merged into operational taxonomic units (OTUs) using UPARSE 7.1, applying a 97% similarity threshold. The taxonomy of each OTU representative sequence was analyzed using RDP Classifier version 2.2, referring the 16S rRNA gene database with a confidence threshold of 0.7. The metagenomic functions were predicted using Phylogenetic Investigation of Communities by Reconstruction of Unobserved States (PIC-RUSt2) based on OTU representative sequences. This study selected six samples for each group, as previous research indicates that six fecal samples effectively reflect the reproducibility of 16S sequencing (Meng et al., 2022).

### 2.7 The target metabolites-measuring

50 mg faecal samples were thawed (4°C for 30 min) and then placed into a 1.5 ml centrifuge tube. Next,1 ml of a 50% methanol aqueous solution was added into the tube, and the mixture was vortexed for 30 min. The tube was then centrifuged at 4°C and 12,000 rpm for 5 min. The supernatant (50 μl) was combined with 150 μl of internal standards consisting of propionic acid isotope standard solution (50 μl, 5 μg/ml), 3-nitrophenylhydrazine (50 μl, 250 mM) methanol/water (1:1, v/v) solution and 1-(3-dimethylaminopropyl)-3-ethylcarbodiimide hydrochloride (50 μl, 150 mM), the mixture was then mixed at 30°C for 30 min. Afterward, butylated hydroxytoluene methanol solution (50 μl, 2 mg/ml) and methanol/water (250 μl, 3:1, v/v) solution were added into the tube and mixed. The tube was centrifuged at 4°C and 12,000 rpm for 5 min. The supernatant (200 μl) was transferred into the vial for mass spectrometry detection. The detection methods for TMA, TMAO and betaine are idential to those used for the SCFAs described previously. The concentration ranges of detection method was 0.1–10,000 ng/ml.

### 2.8 Statistical analysis

Statistical analyses were performed by Graphpad Prism (version 9.0) and R software (version 4.2.1, http://www.r-project.org). One-way analysis of variance (ANOVA) was used to assess normality with equal variance. Subsequently, Tukey’s multiple comparison test was performed for post-hoc analysis. Welch’s ANOVA was applied to analyze normality with unequal variance, followed by the Games-Howell post-hoc test. Mann–Whitney and Kruskal-Wallis tests were used as nonparametric methods of analysis. Principal coordinate analysis (PCoA) using the Bray-Curtis distance matrix was performed in R (packages: ggplot2) to compare the differences in microbial community structure. PERMANOVA based on the Bray-curtis distance matrix was employed in R (package: vegan, adonis function) to assess the significant differences in the gut microbiome. Linear discriminant analysis effect size (LEfSe) was conducted to identify potential indicator species. This was done using the Kruskal–Wallis (KW) sum-rank test to examine core bacterial phenotype across the three groups, with a signidiant threshold of *p* < 0.05 and a LDA score ≥ 4.0. Spearman coefficients were calculated in R (package: corrplot, ggplot2) to reveal correlations between gut microbiota and the target metabolites. All of the *p* value was utilized Bonferroni or FDR correction. All statistical differences in this study were considered significant at *p* < 0.05.

## 3 Results

### 3.1 O3-RI delayed the progression of atherosclerosis of mice

**Figure 1:**
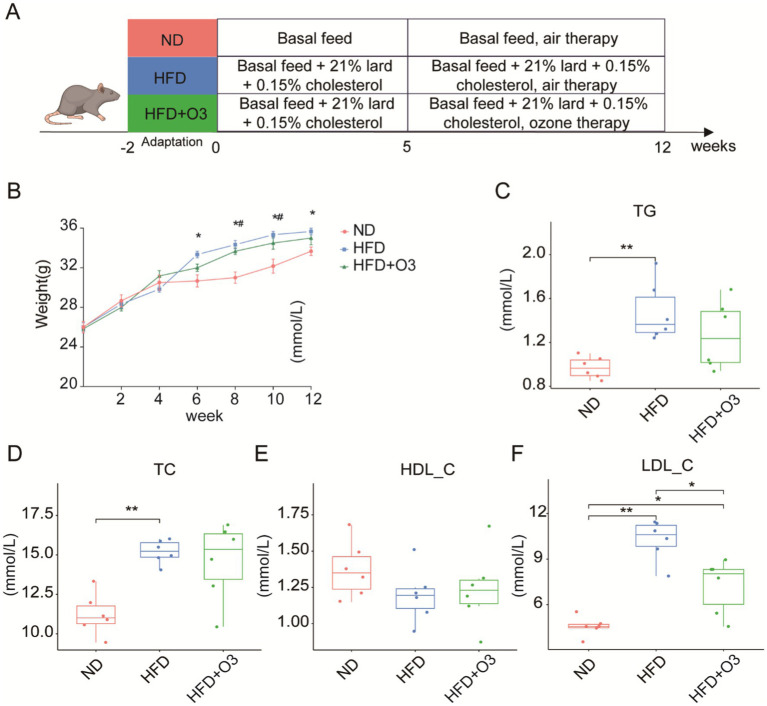
O3-RI improved the body condition and decreased the serum LDL-C level of in HFD mice. **(A)** Design of atherosclerosis model in mice induced by high fat diet. **(B)** The body weights of the mice (*n* = 6). The body weights were significantly different in the HFD group from the 6th to 12th week compared to the ND group, *p < 0.05 compared to the NP group. The body weights significantly increased in the HFD + O3 group from the 8th to 10th week compared to the ND group, ^#^*p* < 0.05 compared to the NP group. The levels of serum TG **(C)**, TC **(D)**, HDL-C **(E)**, and LDL-C **(F)**. *p < 0.05, **p < 0.01.

After a 2-week acclimatization period, the ApoE−/− mice were randomly assigned to three groups: normal diet (ND), high-fat diet (HFD) and HFD with ozone therapy (HFD + O3) groups (Figure 1A). There were no significant differences in food or water consumption among the groups. The body weight of HFD group increased significantly from the 6^th^ to the 12^th^ week, distinguishing it from the ND group. Following the O3-RI intervention with a does of 1 mg/kg(bw), the body weight of HFD + O3 group significantly increased from weeks 8 to 10, differing from the ND group, and there was no difference from week 10 to 12 (*p* < 0.05, Figure 1B). All of the mice were in good condition except that the mice in HFD group exhibited immobile. These results indicated that ozone therapy may improved the body condition of the HFD mice. After 12 weeks of feeding, serum lipid levels were evaluated. The results showed that TG, TC, and LDL-C levels in the HFD group were significantly higher than that in the ND group, LDL-C levels in the HFD + O3 group were substantially lower by about 29.6% than those in the HFD group (*p* < 0.05, Figures 1C,D,F), in contrast, HDL-C levels did not differ significantly among the three groups (*p* < 0.05, Figure 1E).

**Figure 2:**
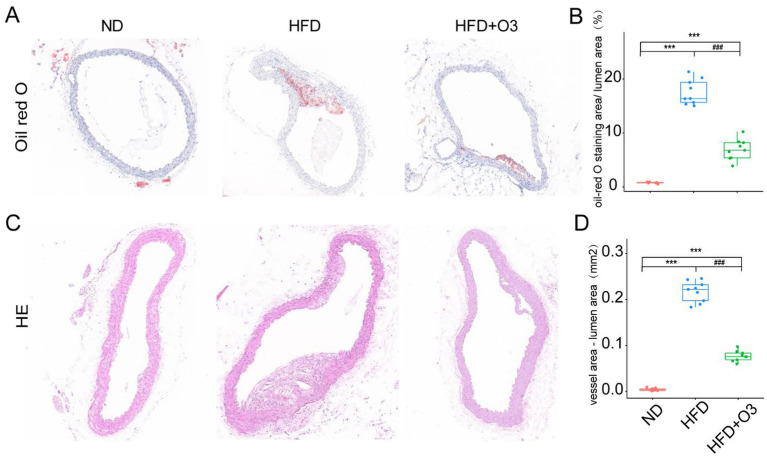
Representative images of oil-red O and HE staining in the aortic sinus and thoracis aorta of the three groups. **(A)** The atherosclerotic lipids area significantly increased in the HFD group compared to the ND group, however the lipids area decreased in the HFD + O3 group. **(B)** The lipid area was quantified by using Image J software in the three groups. *n* = 3, 2-3section/ aortic sinus and thoracis aorta/ group. **(C)** The atherosclerotic plaque area significantly increased in the HFD group compared to the ND group, however the plaque area decreased in the HFD + O3 group. **(D)** The plaque area was quantified by using Image J software in the four groups. *n* = 3, 2-3section/ aortic sinus and thoracis aorta/ group. ***p < 0.001, compared to the ND group. ^###^*p* < 0.001, com-pared to the HFD group; O3-RI decreased the atherosclerotic lipids area during the development of the atherosclerosis.

The aortic tissues were stained with Oil red O and quantified using Image J software(Figure 2A). The atherosclerotic lipid area in the HFD group was larger than that in the ND group. The atherosclerotic lipid area in the HFD + O3 was significantly smaller than in the HFD group (Figure 2B). These findings suggest that O3-RI inhibited the development of atherosclerosis. The aortic tissues were stained with HE and quantified by calculating the difference between the vessel and lumen areas (Figure 2C). These results confirmed the findings obtained with Oil red O staining. We found that in the HFD group, the atherosclerotic plaque area was significantly larger than that in the ND group (*p* < 0.001, Figure 2D). In the HFD + O3 group, the atherosclerotic plaque area was smaller than in the HFD group (*p* < 0.001, Figure 2D). These results suggested that ozone therapy could effectively slow the progression of atherosclerosis.

### 3.2 O3-RI remodeled the gut microbiota of HFD mice

**Figure 3:**
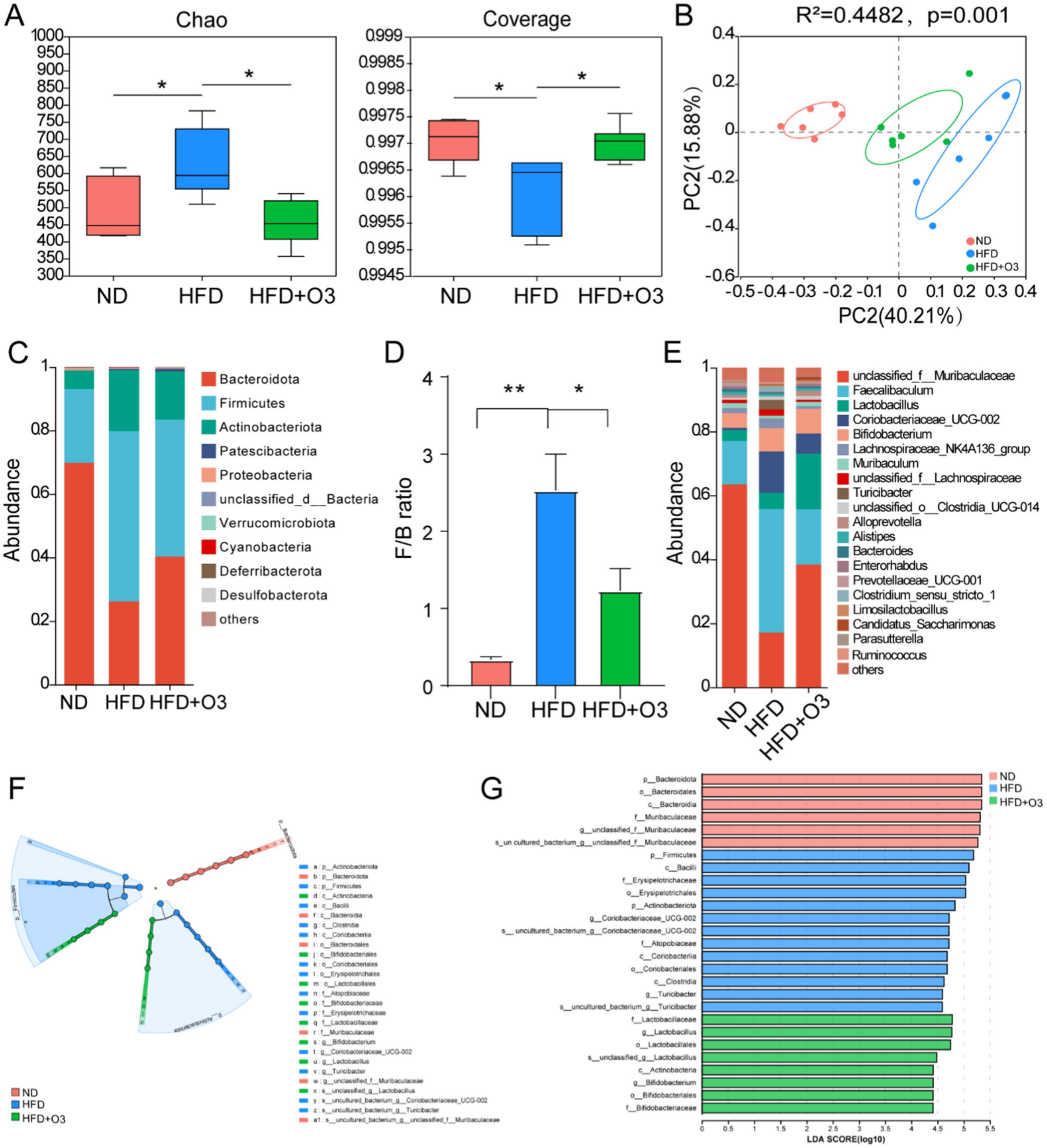
O3-RI regulated the dysbiosis gut microbiota in the high fat diet feeding ApoE−/− mice. **(A)** α diversity index of gut microbiota in mice, *p < 0.05. **(B)** Principal coordinate analysis (PCoA) of microbiota community based on Bray-curtis distance at the operational taxonomic unit (OTU) level. **(C)** Relative abundance of gut microbiota at the phylum level. **(D)** The ratio of Firmicutes/Bacteroidetes in phylum level. **(E)** Relative abundance of gut microbiota at the genus level. *n* = 6. **(F)** LEfSe analysis showed that the taxa of the three groups were significantly enriched. Only taxa with LDA (Linear discriminant analysis) scores of more than 4 were presented. Each ring represents the level of taxa from phylum to species. The diameter of each point on the ring represents the relative abundance of the taxon. **(G)** LDA discriminatory results of LEfSe analysis. LDA > 4, *n* = 6.

The gut microbiota is an important factor in the development of atherosclerosis. 16S RNA-sequencing was conducted to explore how O3-RI affects the structure and composition of gut microbiota. Eighteen faecal samples from the three groups were analyzed, resulting in a total of 6,265 OTUs obtained through data processing. The Chao1 index was conducted to assess the abundance of the microbial community, and the Coverage index was performed on the coverage of the sample database. The α-diversity results indicated that Chao1 and Coverage in HFD group were significantly different from those in ND group. In the HFD + O3 group, these indices were restored, indicating that ozone thrapy enhances the richness and diversity of gut microbiota (Figure 3A). To further investigate, we employed Principal coordinate analysis (PCoA) to reveal differences in the composition of bacterial communities. PCoA results at both OTUs level revealed that high-fat diet significantly affected bacterial community composition (PERMANOVA: R^2^ = 0.4482, *p* = 0.001; Figure 3B) in HFD mice. The microbial communities of mice in ND group and HFD group were significantly separated, while the microbial communities of HFD + O3 group were more similar to the ND group (Figure 3B). These results indicate that ozone treatment can effectively remodel the dysbiotic gut microbiota of HFD mice.

The results showed that the gut microbiota varied significantly at different taxonomic levels among the three groups. *Bacteroidetes*, *Firmicutes*, *Actinobacteriota*, *Proteobacteria* and *unclassified_d__Bacteria* were the main bacteria phyla, and the microbiota composition varied notably among the three groups(Figure 3C). The proportion of *Bacteroidetes* in the HFD group was significantly lower compared to the ND group, while O3-RI intervention led to an increase in the proportion of *Bacteroidetes*. In comparison to the ND group, the abundance of *Firmicutes* in the HFD group markedly increased, while O3-RI intervention decreased the abundance of *Firmicutes*. The *Firmicutes*/*Bacteroidetes* (F/B) ratio was higher in the HFD group was higher than in the ND group, but the O3-RI intervention reduced this ratio (Figure 3D). The relative abundance of microbiota varied significantly among the three groups at the genus level. In the HFD group, the relative abundance of *Muribaculaceae* and *Enterorhabdus* decreased, whereas the abundance of *Faecalibaculum*, *Coriobacteriaceae_UCG-002* and *Turicibacter* increased compared to the ND group. O3-RI intervention altered the gut microbiota composition by increasing the relative abundance of *Muribaculaceae*, *Enterorhabdus* and *Lactobacillus*. Meanwhile, O3-RI decreased the relative abundance of *Faecalibaculum*, *Coriobacteriaceae_UCG-002*, and *Turicibacter* (Figure 3E).

We evaluated the dominant bacterial communities from phylum to genus in the microbiota composition of the three groups using the Linear Discriminant Analysis Effect Size (LEfSe). The core bacterial community in ND group included *Muribaculaceae*, *Bacteroidota*, *Bacteroida*, and *Bacteroidalees*, while the HFD group were enriched with *Actinobacteriota*, *Firmicutes*, *Clostridia*, *Bacilli*, *Coriobacteriales*, *Erysipelotriachales*, *Coriobacteriaceae_UCG-002*, and *Turicibacter* (Figures 3F,G). The results demonstrated that HFD altered the gut microbiota composition compared to the ND group. The HFD + O3 group showed an increase in *Actinobacteria*, *Bifidobacteriales*, *Lactobacillales*, *Bifidobacteriaceeeae*, *Lactobacillaceae*, and *Lactobacillus*.

### 3.3 Enrichment of metabolic and signaling pathways of different microbial communities

**Figure 4:**
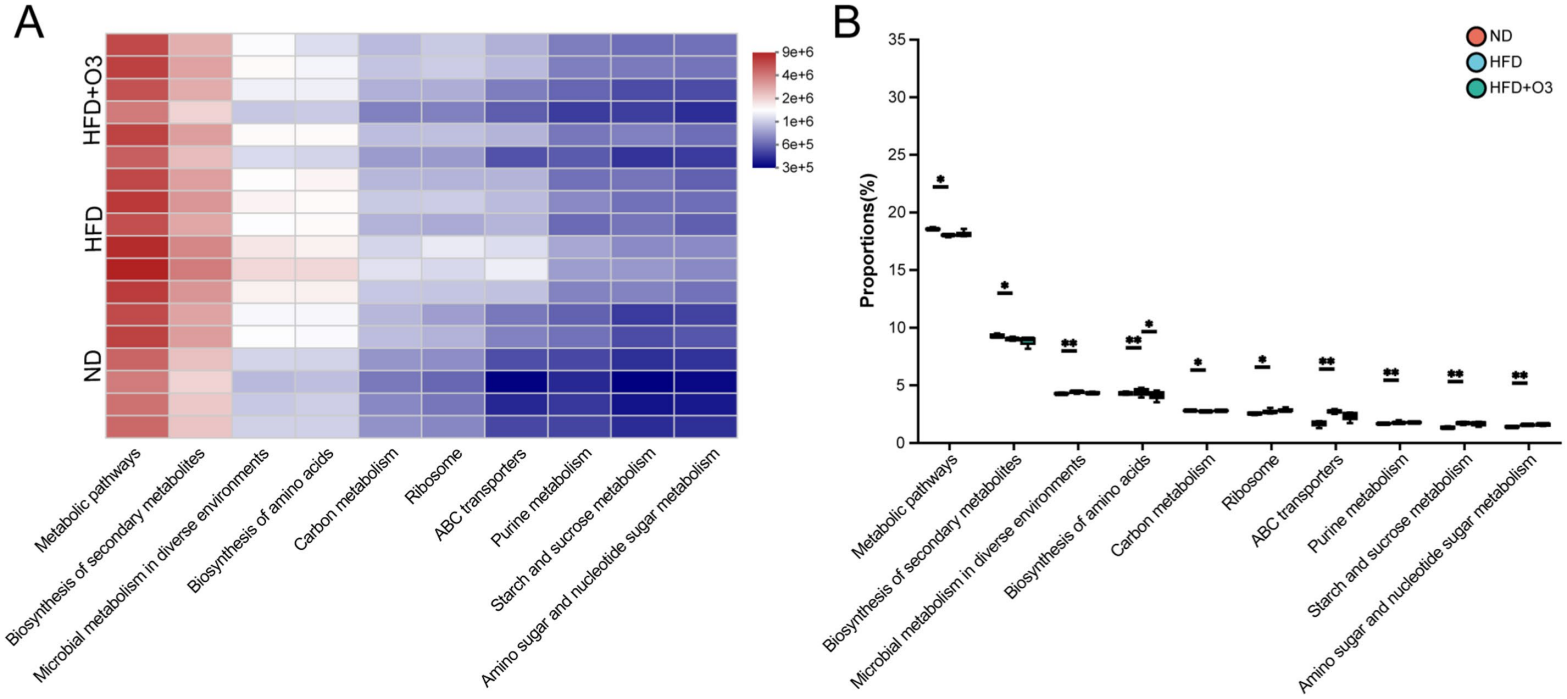
The Kyoto Encyclopedia of Genes and Genomes (KEGG) pathway analyzed the path-ways that showed difference abundances among the three groups. **(A)** The KEGG analyzed the heatmap of pathway Level 3 and showed top 10 pathway in total abundance. **(B)** The 10 path-ways were analyzed statistically among the three groups, *n* = 6.

The Kyoto Encyclopedia of Genes and Genomes (KEGG) pathway from PICRUSt2 showed differences metabolic pathways among the three groups, including secondary metabolites biosynthesis, microbial metabolism, amino acids biosynthesis, carbon metabolism, ribosomal functions, ABC transporters, purine metabolism, starch and sucrose metabolism and amino sugar and nucleotide sugar metabolism. HFD feeding ApoE−/− mice can affect the pathways related to gut microbiota metabolism, but O3-RI intervention can regulate these pathways, particularly in the biosynthesis of amino acids (Figure 4).

### 3.4 O3-RI modulated the target metabolites in feces

**Figure 5:**
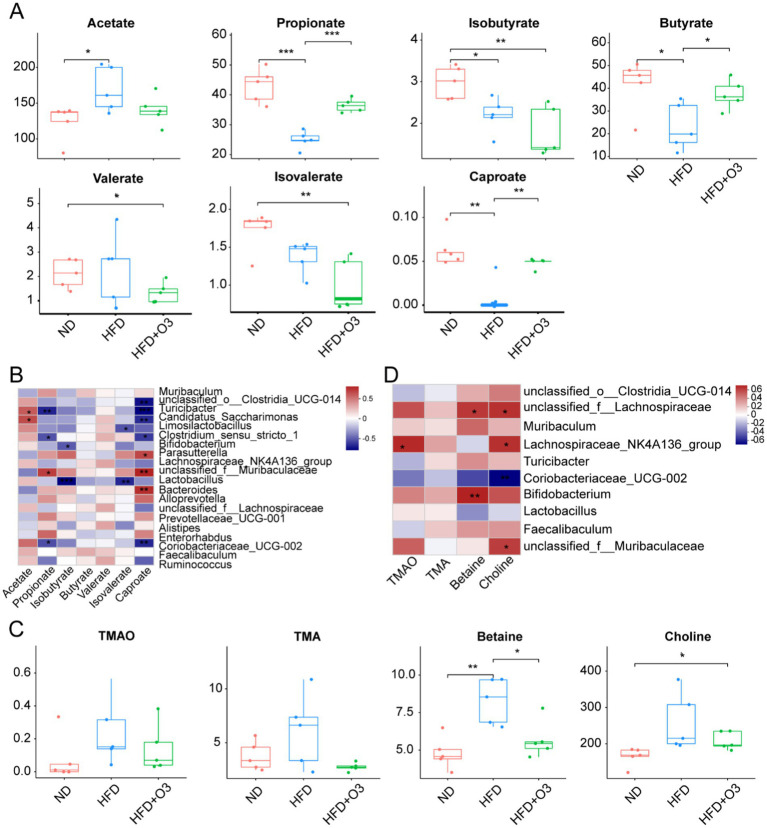
O3-RI modulated the target metabolites. **(A)** The levels of acetate, propionate, isobutyrate, butyrate, isovalerate, valerate, and caproate., compared with HFD group. **(B)** Spearman correlation analysis was conducted to analyze the relationship between gut microbiota and SCFAs in ApoE−/− mice. **(C)** The levels of TMAO, TMA, choline, betaine by LC–MS. **(D)** Spearman correlation analysis was conducted to analyze the relationship between gut microbiota and the target metabolites in ApoE−/− mice. *n* = 5/group. *p < 0.05, **p < 0.01, ***p < 0.001.

SCFAs are crucial in the development of atherosclerosis. LC–MS detection was used to detect these metabolites in the gut microbiota. The result showed that HFD increased acetate levels while reducing propionate, isobutyrate, butyrate, isovalerate and caproate levels in the ApoE−/− mice (Figure 5A). O3-RI modulated SCFAs and inhibited atherosclerosis development. This resulted in decreased acetate levels and increased levels of propionate, butyrate and caproate in the HFD-fed ApoE−/− mice. We conducted a correlation analysis to examine the relationship between gut microbiota and SCFAs in ApoE−/− mice (Figure 5B). The results showed that the abundance of *Muribaculaceae* was positively correlated with propionate and caproate, while the abundance of *Lactobacillus* was negatively correlated with isobutyrate and isovalerate. The correlation between SCFAs and different gut microflora can be analyzed using a correlation heat map.

Many studies showed a positive relationship between Trimethylamine N-oxide (TMAO) levels and atherosclerosis development. TMAO is formed primarily forms when intestinal microflora metabolize nutrient substrates in the colon. These substrates include phosphatidylcholine/choline, carnitine, betaine, dimethylglycine and ergothioneine. LC–MS detection was used to measure the level of TMAO, TMA, betaine and choline. The result indicated that HFD increased TMAO, TMA, betaine and choline levels in ApoE−/− mice, while O3-RI effectively lowered these metabolites (Figure 5C). O3-RI can significantly reduce the concentration of betaine compared to the HFD group. The correlation analysis was conducted to study the relationship between gut microbiota and these target metabolites in ApoE−/− mice (Figure 5D). Correlation results revealed that the abundance of *Muribaculaceae* was negatively correlated with choline, whereas *Turicibacter* and *Coriobacteriaceae_UCG-002* was positively correlated with betaine. Additional, the abundance of *Lactobacillus*, *Bifidobacterium*, and *coriobacteriaceae_UCG-002* showed a positive correlation with choline.

## 4 Discussion

Estrogen has anti-inflammatory and vascular protective effects that can slow down the onset and progression of atherosclerosis (Yerly et al., 2023). We used male ApoE−/− C57BL/6 mice to induce atherosclerosis by feeding a high-fat diet in this study. The atherosclerotic lesions in ApoE−/− mice are similar to the developmental and pathological processes of atherosclerosis in humans. We then applied O3-RI with a does of 1 mg/kg(bw) to treat atherosclerosis and investigated the potential mechanism for inhibiting disease progression. We observed changes in serum lipid levels and alterations in the composition and structure of gut microbiota, including SCFAs which are the target metabolites. In addition, we examined how SCFAs correlate with gut microbiota. Our findings suggested that O3-RI could prevent and inhibit the development of atherosclerosis by intervening in the formation of arterial lipids and plaques. Specifically, O3-RI was linked to lower LDL-C levels. Furthermore, O3-RI seemed to influende the gut microbiota and SCFAs and metabolites involved in atherosclerosis development. O3-RI increased the abundance of probiotics like *Bifidobacterium* and *Lactobacillus* and increased the concentration of propionate, butyrate and caproate acid in the HFD-fed ApoE−/− mice. O3-RI could reduce the levels of TMAO, TMA, betaine and choline, with a significant effect on betaine levels. Notably, the SCFAs and target metabolites in the treatment groups exhibited a strong correlation with both the gut microbiota and the progression of atherosclerosis. These results suggest that O3-RI targeting the gut microbiota and its metabolites may play a role in the prevention, management, and treatment of atherosclerosis.

Atherosclerosis is a progressive chronic inflammatory disease that involves multiple cell types (inflammatory and smooth muscle cells and their secretions) and molecular processes. This disease is characterized by the formation of lipid plaques containing lipid deposits (Aluganti Narasimhulu et al., 2016; Li et al., 2022). Ozone therapy is widely used to treat vascular system diseases, including severe peripheral artery disease, cholesterol embolism, atherosclerosis, and severe dyslipidemia owing to its antioxidant effects (Di Paolo et al., 2005; Orakdogen et al., 2016). Ozone has been demonstrated to inhibit arterial plaque formation by reducing damaged biomolecules and enhancing antioxidant activity (Delgado-Roche et al., 2013). It has been reported that ozone intervention at appropriate concentrations, mainly particularly in low to medium concentrations (5-35 μg/ml), positively influences the function of endothelial cells (Cenci et al., 2022; Orakdogen et al., 2016). In our *in vitro* experiments, we incubated endothelial cells with O3 and found that a concentration of 10-15 μg/ml was most effective in promoting endothelial cell proliferation. In our *in vivo* pre-experiments, we found that a concentration of 20 μg/ml was more effective than 10 μg/ml in modulating the gut microbiota in mice with cognitive impairment induced by chronic rapid eye movement sleep deprivation (Cheng et al., 2024). In our study, O3-RI significantly reduced serum level of LDL-C. This treatment improved the pathological and ultrastructural changes in the aortic sinus and thoracic aorta, as assessed by Oil red O and HE staining in HFD-fed ApoE−/− mice. These findings are consistent with the performance of ozone therapy in clinical studies (Kato et al., 2022; Valdes et al., 2018). Ozone intervention could significantly reduce serum cholesterol and LDL-C levels in patients with heart disease or kidney dialysis. However, the mechanism by which ozone lowers cholesterol and LDL-C levels remains unclear. Some scholars attribute this effect to ozone reaction products that influence oxysterols OSTs and antioxidant enzymes, potentially stimulating the lipid synthesis and oxidation (Hernández et al., 1995; Tylicki et al., 2004).

In-depth studies of gut microbiota reveal its crucial role in both physiological and pathological conditions of the body. Numerous studies have shown that changes in the composition and function of the gut microbiota are closely associated with cardiovascular disease (Tang et al., 2017). Studies have indicated that a decline in gut microbiota diversity and abundance may raise the risk of atherosclerosis development (Pieczynska et al., 2020). Our results showed that the HFD increased the richness of gut microbiota while simultaneously reducing its coverage, which contradicts previous studies. Furthermore, O3-RI decreased the diversity and richness of gut microbiota while increasing its coverage in ApoE−/− mice. High alpha diversity is often seen as beneficial for health because it may improve digestion, immune system function, and overall metabolic health. However, gut microbiota alpha diversity is not a definitive indicator of health. In fact, increased diversity has been noted in some unhealthy conditions like obesity, dyslipidemia, and hyperinsulinemia (Verhaar et al., 2020). The higher alpha diversity in the gut microbiota of the ApoE−/− mice on a high-fat diet may relate to variations in the diet’s composition and their rearing environment, among other factors. PCoA indicated that the HFD group was distinct from the ND group, indicating that high-fat diet significantly altered the structure and composition of the gut microbiota. O3-RI intervening altered the overall structure and composition of the gut microbiota, making it more similar to that of the ND group. This suggests that O3-RI effectively reshaped the microflora structure and composition in the ApoE−/− mice.

The most abundant phyla in the human gut microbiota are Firmicutes and BacteroidetesCardiovascular diseases are closely associated with changes in composition of bacterial community across various taxonomic levels. The most abundant phyla in the human gut microbiota are *Firmicutes* and *Bacteroidetes* (Ley et al., 2006). Some studies showed that *Bacteroidetes* have an anti-inflammatory effect and reduce the occurrence of atherosclerosis, while *Firmicutes* had the opposite effect (Yamashita, 2017). In our study, the abundance of *Bacteroidetes* was significantly lower in the HFD group than in the ND group. Additionally O3-RI increased the abundance of *Bacteroidetes* in the HFD-fed ApoE−/− mice. Meanwhile, the abundance of *Firmicutes* significantly increased in the HFD-fed ApoE−/− mice, O3-RI reduced the abundance of *Firmicutes*. It was reported that the *Firmicutes* to *Bacteroidetes* ratio was positively associated with cardiovascular diseases (Emoto et al., 2016). This radio increases in obesity, HFD-fed mouse models and even with aging (Magne et al., 2020; Porras et al., 2017; Spychala et al., 2018). In our study the *Firmicutes* to *Bacteroidetes* ratio was higher in the HFD group compared to the ND group. In contrast, O3-RI intervention lowered this ratio. These findings suggest that O3-RI holds promise as a therapeutic approach for prevention the development of atherosclerosis.

Compared to the ND group, HFD reduced the abundance of *Muribaculaceae* and *Enterorhabduse* in mice. *Muribaculaceae* was proved to be negatively correlated with hepatic triglyceride and total cholesterol levels (Yuan et al., 2021). A higher abundance of *Enterorhabduse* improves the intestinal capacity to combat oxidative stress (Gao et al., 2023). These changes likely contribute to the promotion of atherosclerosis by HFD. After O3-RI treatment, the abundance of these microorganisms increased again. Furthermore, the O3-RI intervention modified the microbiota composition at the genus level, resulting in an increased the abundance of *Lactobacillus* and *Bifidobacterium*. *Lactobacillus* and *Bifidobacterium* are both recognized for their ability to significantly reduce LDL cholesterol and total cholesterol. This reduction lowers the risk of cardiovascular disease (Alexandrescu et al., 2024; Chan et al., 2016; Jia et al., 2023; Qiu et al., 2022).

The abundance of *Coriobacteriaceae_UCG-002*, *Turicibacter*, *Clos-tridium_sensu_stricto_1*, *Stenotrophomonas*, and *Lachnospiraceae* was significantly increased in the HFD group. The relationship between *Coriobacteriaceae_UCG-002* and cardiovascular disease is less reported, and its function needs further study. The bacteria of the genus *Turicibacter* are essential members of mammalian gut microflora and are related to dietary fat. Introducing the bile-modifying gene from the *Turicibacter* strain can lower serum cholesterol, triglyceride and adipose tissue in mice (Lynch et al., 2023). It is suggested that *Turicibacter* can protect cardiovascular from hyperlipidemia. Strangely, *Turicibacter* increased in the HFD group but decreased in the HFD + O3 group. A clinical trial indicated that *Clostridium sensu stricto 1* was positively correlated with blood pressure. This study involved 33 subjects who had not been treated with antihypertensive drugs (Huart et al., 2019). After ozone treatment, the abundance of this genus significantly decreased. These results suggest that this genus could serve as a marker of cardiovascular disease. *Lachnoclostridium* is closely related to several specific metabolic diseases. A strain of *Lachnoclostridium* can catalyze choline to produce trimethylamine (TMA). This process promotes the development of atherosclerosis (Cai et al., 2022). Furthermore, HFD increased the abundance of *Lachnospiraceae,* raising the risk of atherosclerosis. Conversely, ozone therapy has bebn shouwn to restore the abundance of these bacteria.

TMAO, a significant contributor to cardiovascular disease among microbial metabolites, has garnered considerable attention. TMAO isproduced in the liver by oxidazion TMA, which mainly comes from the bacterial metabolism of dierary choline and phosphatidylcholine (Bennett et al., 2013; Tang et al., 2013). In mice experiments, the elimination of TMA lyase can significantly inhibit choline metabolism, thereby reducing TMAO production and the development of atherosclerosis (Wang et al., 2015). *γ*-Butyryl betaine is an intermediate metabolite produced of gut microbiota that converts L-carnitine into TMAO, thereby promoting atherosclerosis (Koeth et al., 2014). HFD increased the level of TMAO, TMA, betaine and choline in ApoE−/− mice, while O3-RI reduced these levels back to normal. Moreover, O3-RI could significantly reduce the concentration of betaine.

SCFAs, including acetate, propionate and butyrate, are important metabolites of gut microbiota. It plays a vital role in maintaining intestinal homeostasis (Brown and Hazen, 2018). Research has investigated the cardiovascular protective effects of propionate. In hypertensive mouse models, oral supplementation of propionic acid can significantly reduce cardiac hypertrophy, fibrosis, vascular dysfunction and hypertension. It also lowers susceptibility to ventricular arrhythmia and atherosclerotic lesions (Bartolomaeus et al., 2019). Both propionate (Haghikia et al., 2022) and butyrate (Du et al., 2020; Kasahara et al., 2018) can reduce the atherosclerotic phenotype of ApoE−/− mice induced by HFD. Our results showed that HFD raised acetate levels while lowering the levels of propionate, isobutyrate, butyrate, isovalerate and caproate in the ApoE−/− mice. In contrast, O3-RI reduced acetate levels while increasing propionate, butyrate and caproate levels. Numerous bacteria, including *Clostridium butyricum*, *Bifidobacterium*, *Lactobacillus*, *Bacteroidetes*, *Faecalibacterium* and *Roseburia,* are recognized as SCFA producers (Alexandrescu et al., 2024; Zhao et al., 2022). O3-RI intervention increased the relative abundance of *Lactobacillus* and *Bifidobacterium*, which in turn enhanced SCFAs production in feces.

**Figure 6:**
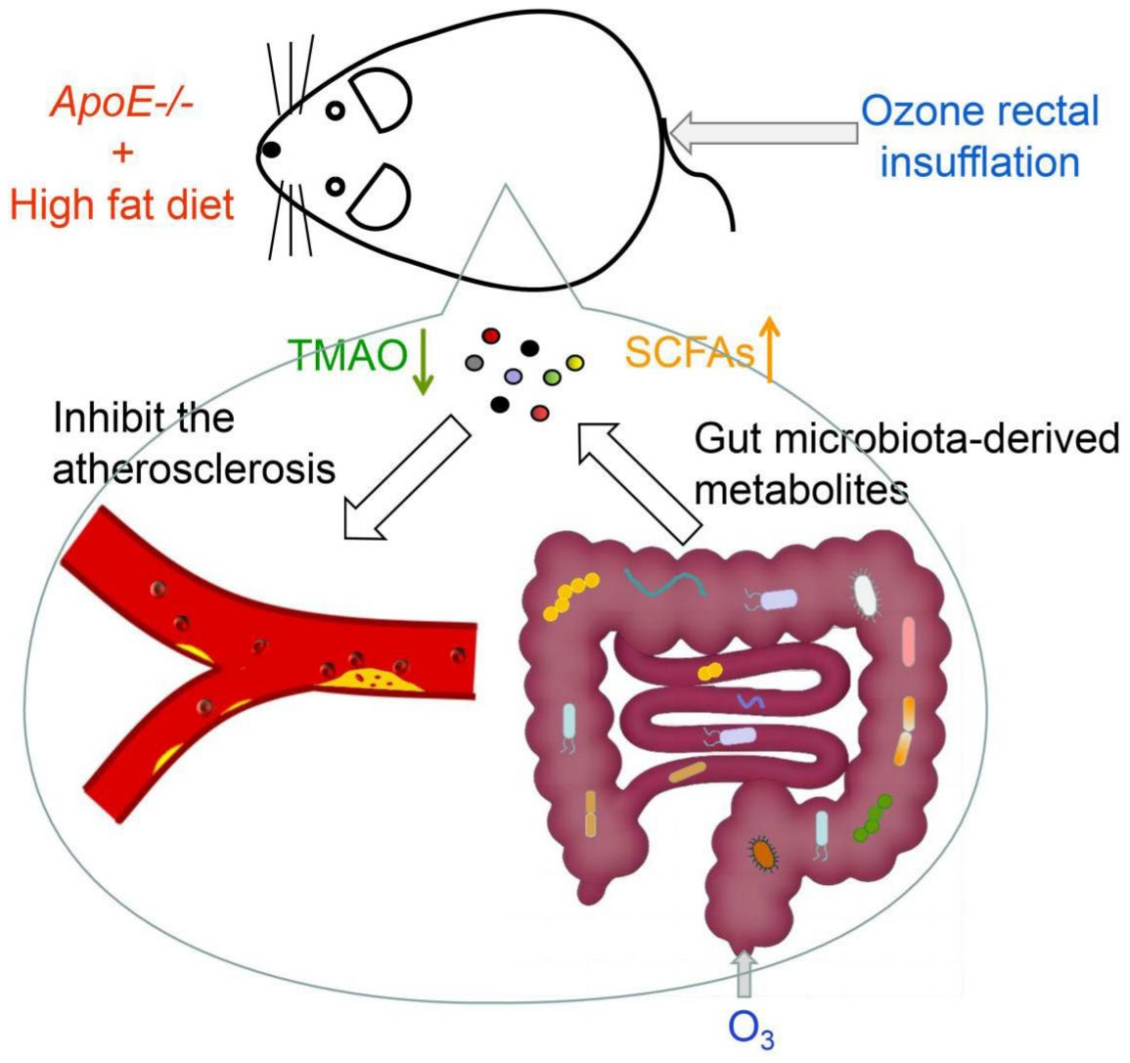
The mechanism of ozone in inhibiting atherosclerosis. O3-RI inhibited atherosclerosis by optimizing gut microbiota and regulating microbial metabolites. O3-RI increased the abundance of beneficial microorganisms and metabolites, including *Lactobacillus*, *Bifidobacterium* and SCFAs, while simultaneously reducing harmful microbial metabolites like TMA and TMAO.

Recently, ozone has become widely used in medicine and has shown promising results in treating various diseases. In our study, we established an atherosclerosis model by feeding male ApoE−/− mice with HFD and demonstrated that O3-RI could inhibit the development of atherosclerosis. The underlying mechanism may involve O3-RI affecting the growth of specific microorganisms, which alters community distribution and ultimately changes metabolic products (Figure 6). Ozone primarily affects microbial growth through oxidative processes, but how it exerts its effects within the animal body still needs further exploration. Compared to conventional drug treatments and interventional therapies, ozone therapy has characteristics such as being painless, non-invasive, and having minimal side effects. This facilitates the use of ozone in the treatment of cardiovascular diseases. Additionally, the safety and simplicity of ozone treatment also make it widely applicable in the care and home health management of patients with cardiovascular diseases.

### 4.1 Limitation

Atherosclerosis is a long-term process, and our experimental study focuses on the early stage. In future experiments, we will conduct studies on long-term development of atherosclerosis and the associated sex differences. We need to further explore how ozone regulates gut microbiota andthe progression of atherosclerosis.

## 5 Conclusion

Atherosclerotic cardiovascular diseases are the leading cause of death. It is important to Identifying targeted therapies to prevent or slow the development of atherosclerosis is crucial. This study demonstrates that O3-RI lowers serum LDL-C levels and reduces the atherosclerotic lipid area and plaque area in ApoE−/− mice. These effects may be associate with O3-RI optimizing gut microbiota and regulating microbial metabolites. O3-RI increased the abundance of beneficial microorganisms and metabolites, including *Lactobacillus*, *Bifidobacterium* and SCFAs, while simultaneously reducing harmful microbial metabolites like TMA and TMAO. Our findings offer new insights into the clinical application of ozone therapy for atherosclerosis, although further research is needed to elucidate the underlying mechanisms.

## Data Availability

The raw sequence data reported in this paper have been deposited in the Genome Sequence Archive in National Genomics Data Center, China National Center for Bioinformation/Beijing Institute of Genomics, Chinese Academy of Sciences (GSA: CRA023854) that are publicly accessible at https://ngdc.cncb.ac.cn/gsa.
